# Target 2035 – an update on private sector contributions

**DOI:** 10.1039/d2md00441k

**Published:** 2023-03-16

**Authors:** Suzanne Ackloo, Albert A. Antolin, Jose Manuel Bartolome, Hartmut Beck, Alex Bullock, Ulrich A. K. Betz, Jark Böttcher, Peter J. Brown, Menorca Chaturvedi, Alisa Crisp, Danette Daniels, Jan Dreher, Kristina Edfeldt, Aled M. Edwards, Ursula Egner, Jon Elkins, Christian Fischer, Tine Glendorf, Steven Goldberg, Ingo V. Hartung, Alexander Hillisch, Evert Homan, Stefan Knapp, Markus Köster, Oliver Krämer, Josep Llaveria, Uta Lessel, Sven Lindemann, Lars Linderoth, Hisanori Matsui, Maurice Michel, Florian Montel, Anke Mueller-Fahrnow, Susanne Müller, Dafydd R. Owen, Kumar Singh Saikatendu, Vijayaratnam Santhakumar, Wendy Sanderson, Cora Scholten, Matthieu Schapira, Sujata Sharma, Brock Shireman, Michael Sundström, Matthew H. Todd, Claudia Tredup, Jennifer Venable, Timothy M. Willson, Cheryl H. Arrowsmith

**Affiliations:** a Structural Genomics Consortium, University of Toronto Toronto Ontario M5G 1L7 Canada suzanne.ackloo@utoronto.ca aled.edwards@utoronto.ca Cheryl.Arrowsmith@uhnresearch.ca; b ProCURE, Catalan Institute of Oncology, Oncobell, Bellvitge Institute for Biomedical Research (IDIBELL) L'Hospitalet del Llobregat Barcelona Catalonia Spain aantolin@idibell.cat; c A Division of Janssen-Cilag S.A., Janssen Research and Development Toledo Spain jbartolo@its.jnj.com JLlaveri@ITS.JNJ.com; d Research and Development, Bayer AG, Pharmaceuticals 42103 Wuppertal Germany hartmut.beck@bayer.com jan.dreher@bayer.com alexander.hillisch@bayer.com; e Center for Medicines Discovery, Old Road Campus, University of Oxford Roosevelt Drive, Headington Oxford OX3 7DQ UK alex.bullock@cmd.ox.ac.uk jon.elkins@cmd.ox.ac.uk; f Merck KGaA Darmstadt Germany ulrich.betz@merckgroup.com Sven.Lindemann@merckgroup.com; g Boehringer Ingelheim RCV GmbH & Co KG Vienna Austria jark.boettcher@boehringer-ingelheim.com; h Structural Genomics Consortium, University of North Carolina at Chapel Hill USA peter.brown@unc.edu; i Boehringer Ingelheim International Binger Str. 173 D-55216 Ingelheim Germany menorca.chaturvedi@boehringer-ingelheim.com markus.koester@boehringer-ingelheim.com oliver.kraemer@boehringer-ingelheim.com; j Division of Cancer Therapeutics, The Institute of Cancer Research London SM2 5NG UK Alisa.crisp@icr.ac.uk; k Foghorn Therapeutics 500 Technology Square, Suite 700 Cambridge MA 02139 USA DDaniels@foghorntx.com; l Structural Genomics Consortium, Department of Medicine, Karolinska University Hospital and Karolinska Institutet Stockholm Sweden kristina.edfeldt@ki.se michael.sundstrom@ki.se; m Nuvisan Innovation Campus Berlin GmbH Müllerstraße 178 13353 Berlin Germany egner.target2035@web.de anke.mueller-fahrnow@nuvisan.com; n Discovery Chemistry, Merck & Co., Inc. Boston Massachusetts 02115 USA christian_fischer@merck.com; o Research & Early Development, Novo Nordisk A/S Måløv Denmark tgle@novonordisk.com lrli@novonordisk.com; p Janssen Research and Development LLC San Diego California USA SGoldbe1@its.jnj.com ssharm505@ITS.JNJ.com BShirema@its.jnj.com JVenable@its.jnj.com; q Medicinal Chemistry, Global R&D, Merck Healthcare KGaA Frankfurter Straße 250 64293 Darmstadt Germany ingo.hartung@merckgroup.com; r Science for Life Laboratory, Department of Oncology-Pathology, Karolinska Institutet Stockholm Sweden evert.homan@ki.se maurice.grube@scilifelab.se; s Institute of Pharmaceutical Chemistry, Goethe University Frankfurt Frankfurt 60438 Germany knapp@pharmchem.uni-frankfurt.de tredup@pharmchem.uni-frankfurt.de; t Structural Genomics Consortium, BMLS, Goethe University Frankfurt Frankfurt 60438 Germany susanne.mueller-knapp@bmls.de; u Boehringer Ingelheim Pharma GmbH & Co. KG Birkendorfer Str. 65 D-88397 Biberach an der Riss Germany uta.lessel@boehringer-ingelheim.com; v Neuroscience Drug Discovery Unit, Research, Takeda Pharmaceutical Company Limited Fujisawa Kanagawa Japan hisanori.matsui@takeda.com; w Boehringer Ingelheim Pharma GmbH & Co. KG Birkendorfer Str. 65 D-88397 Biberach an der Riss Germany florian.montel@boehringer-ingelheim.com; x Discovery Network Group, Pfizer Medicine Design Cambridge MA 02139 USA Dafydd.Owen@pfizer.com; y Global Research Externalization, Takeda California, Inc. 9625 Towne Center Drive San Diego CA 92121 USA kumar.saikatendu@takeda.com; z Janssen Research & Development, Janssen Pharmaceutica N. V Beerse Belgium WSANDERS@its.jnj.com; a Research and Development, Bayer AG, Pharmaceuticals 13353 Berlin Germany cora.scholten@bayer.com; b Department of Pharmacology & Toxicology, University of Toronto Toronto Ontario M5S 1A8 Canada; c School of Pharmacy, University College London London WC1N 1AX UK mattoddchem@gmail.com; d Structural Genomics Consortium, UNC Eshelman School of Pharmacy, University of North Carolina at Chapel Hill Chapel Hill NC 27599 USA tim.willson@unc.edu; e Princess Margaret Cancer Centre Toronto Ontario M5G 1L7 Canada

## Abstract

Target 2035, an international federation of biomedical scientists from the public and private sectors, is leveraging ‘open’ principles to develop a pharmacological tool for every human protein. These tools are important reagents for scientists studying human health and disease and will facilitate the development of new medicines. It is therefore not surprising that pharmaceutical companies are joining Target 2035, contributing both knowledge and reagents to study novel proteins. Here, we present a brief progress update on Target 2035 and highlight some of industry's contributions.

## Abstract 2

Target 2035, an international federation of biomedical scientists from the public and private sectors, is leveraging ‘open’ principles to develop a pharmacological tool for every human protein.

## Graphical abstract

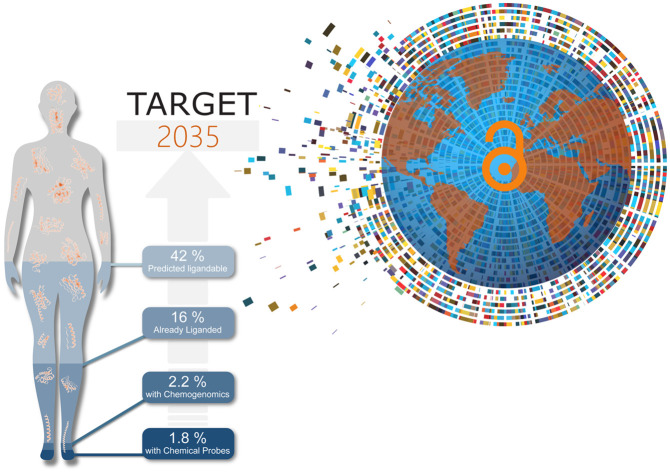

## Introduction

**Fig. 1:**
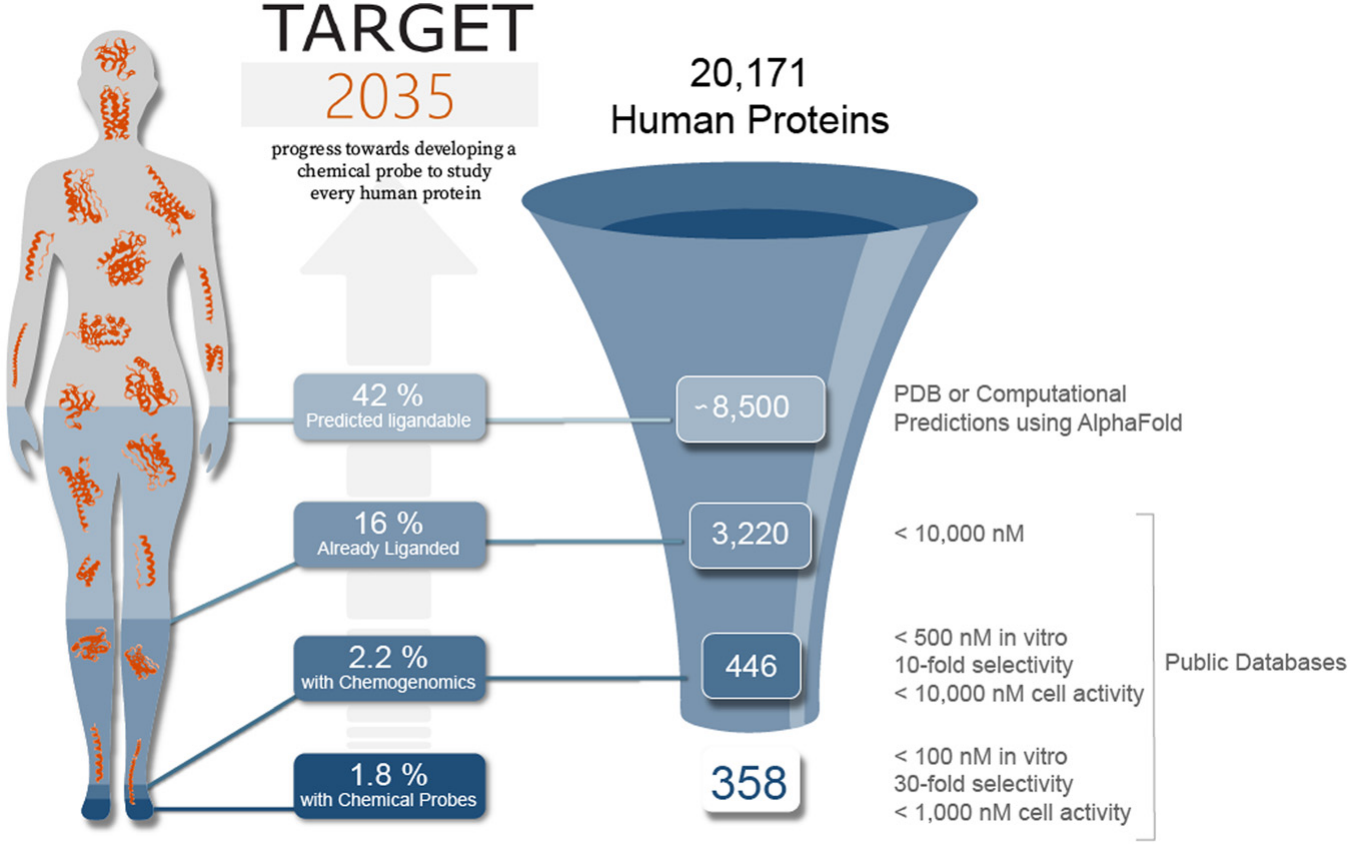
Current analysis of the global scope of chemical probes. The percentage of the proteome that is predicted to be ligandable has been derived from canSAR;^24^ it calculates the ligandability of all proteins using 3D structures from either the PDB and/or AlphaFold Protein Structure Database. The liganded proteome has been calculated assuming that a protein is liganded if it has a small molecule in public chemical databases^24^ with a binding or inhibition activity more potent than 10 micromolar. The human targets that can be currently studied with a chemogenomics library or a chemical probe have been calculated from public databases^24^ using the potency, selectivity and cell activity thresholds displayed. This analysis does not include data from patents unless they have been curated in ChEMBL^25^ or BindingDB.^26^

Formulated in 2018, by public funders and scientific representatives from academia and the drug discovery industry, Target 2035 is focused on developing a pharmacological tool for each human protein by the year 2035.^1–5^ This is a significant challenge as illustrated by the current view of the liganded proteome (Fig. 1) especially relative to Antolin *et al.*'s assessment of the liganded proteome in 2018.^6^ We are, however, confident that, with open and unrestricted science and a global federated effort from academia and industry, we will meet this challenge.^7–14^ Target 2035 aims to create an open and collaborative ecosystem through forming public–private partnerships (PPPs), hosting virtual events *via*https://target2035.net,^15^ open science communities such as Just One Giant Lab,^16^ and contributing to the organization of in-person workshops such as at the International Chemical Biology Society conference (https://icbs2022.chemical-biology.org/).

The goals of Target 2035 can only be accomplished by expanding hit-finding approaches beyond traditional methods. This is especially true for un/understudied proteins that have no link to disease. Traditional hit-finding methods tend to be prohibitively resource-intensive and limited to large private sector entities. During the past year Target 2035 launched two PPPs, Critical Assessment of Computational Hit-finding Experiments (CACHE),^17^ and Open Chemistry Networks (OCN).^18^ CACHE was conceived by a working group of experts from the public and private sector, including leaders of D3R, a previous similar initiative.^19^ Because CACHE is a prospective hit-finding exercise, where predicted compounds are procured and tested using biophysical and biochemical methods, the metric of success is hit rate, diversity, and drug likeness rather than binding pose or docking score. As such, CACHE is complementary to previous and current excellent computational chemistry benchmarking efforts such as D3R or SAMPL.^19,20^

In CACHE,^17^ computational chemists have three unique opportunities per year to benchmark their algorithms and processes. In every challenge, each participant predicts up to 100 ligands for new, expert-curated targets. The predicted compounds are purchased, evaluated experimentally in target-specific assays, and binding data returned to participants. For methods that successfully predicted binders, a second (and final) round of predictions is performed making use of the experimental results from the first round, if possible. Three benchmarking challenges (https://cache-challenge.org/) are currently underway for LRRK2 WD40 repeat domain (WDR),^21^ SARS-CoV-2 NSP13 RNA-binding domain, and SARS-CoV-2 NSP3 ADP-ribose binding site each with 25 participants.^22^

OCN is an international, open, and inclusive chemistry network that exists to bring together chemists and biochemists in the community with a common aim of developing probes to understudied targets.^23^ It has the potential to involve global chemistry contributors, helping to develop small molecule binders for proteins for which there may be no local expertise. Since all data are open and clearly licenced as CC-BY, contributors can use these data in their own applications for follow-on funding. The current targets include RBBP4, HIPK4, PLCZ1, NSP13, and ABHD2 each having unique needs.^18^ However, all projects are contingent on synthetic chemist trainees, and target champions from the drug discovery industry. The data generated may be used to facilitate funding opportunities for downstream research.

Pharmaceutical companies have collaborated effectively within PPPs, especially in the pre-competitive space^8,27,28^ and will continue to contribute towards Target 2035 by donating^29^ and co-developing chemical probes,^4,30–34^ and other pharmacological tools which can be used by the global research community without restrictions. In addition, industry (outside the umbrella of Target 2035) hosts open innovation programs wherein the global academic research community can access first-in-class compounds, by proposing exciting hypothesis-driven science. Noteworthy examples are the AACR-Bayer Innovation and Discovery Grants,^35^ Boehringer Ingelheim's OpnMe,^7,36,37^ Merck KGaA's Open Innovation Portal,^38^ Co-Create Knowledge for Pharma Innovation with Takeda Funding,^39^ and Novo Nordisk's Compound Sharing platform.^40^ In the following section, representatives from the private sector independently added granularity to their company's contributions to open science.

## Summaries of private sector contributions to Target 2035

### Bayer

Bayer is highly committed to contribute to open science by engaging in joint projects with academia and by donating high-quality chemical probes and chemogenomic libraries.^29^ Thus far, 28 chemical probes made by Bayer have been contributed to open science during long-term collaborations with Structural Genomics Consortium (SGC) and IMI projects, such as EUbOPEN.^41^ The majority are donated probes resulting from dedicated in-house lead optimization programs aiming at clinical candidate compounds, which speaks for their quality and relevance for the scientific community. Bayer's first donated probe in 2015 was the human neutrophil elastase (HNE) inhibitor BAY-678, profiled side by side with its close congener the clinical compound BAY 85-8501 for the treatment of pulmonary diseases.^42^ Bayer's latest donated probe in 2022 is the former clinical candidate compound BAY 1816032, a potent and selective BUB1 inhibitor for cancer https://www.sgc-ffm.uni-frankfurt.de/#!specificprobeoverview/BAY%201816032.

Based on the recognition that “in open science everybody wins”, Bayer is increasingly interested to join efforts with academia to evaluate innovative targets. The idea is to combine the best of both worlds, by bringing together industrial lead optimization expertise with specific academic bioassay knowledge, for the benefit of all parties. Bayer's first co-developed chemical probe was the SMYD2 inhibitor BAY-598 for cancer.^43^ The latest example is the USP21 inhibitor BAY-805^44^ which is the first potent and selective probe for USP21. It will help researchers gain new insights into the intriguing disease biology of immune-oncology.

Apart from providing chemical probes, Bayer is also engaged in a variety of IMI projects contributing to open science *via* developing IT tools, databases, and technology platforms. Bayer shares the opinion that the ambitious goal of the Target 2035 initiative, to deliver a chemical probe for every potential target protein, can only be achieved by enhanced use of computational tools. To support this effort, Bayer co-initiated together with the SGC the CACHE^17^ initiative, to predict small molecules as potential target protein binders and to benchmark and ultimately improve various computational hit-finding methods.

All these manifold tools provided through open science will strongly contribute to deepen the understanding of disease-related biological processes and thus pave the way for the future development of new medicines for patients. Everybody wins.

### Boehringer Ingelheim opnMe

Boehringer Ingelheim is consciously committed to continuously foster innovation through open science. To achieve this goal, we are sharing our best tool compounds and beyond *via* different initiatives: https://www.theSGC.org, https://EUbOPEN.org, and https://opnMe.com.^7,36,37^ The driver behind our open science initiatives is the recognition that, “No matter who you are, most of the brightest brains work for someone else,” and not only in your own organization. Our open science initiatives seek to lift barriers between academic researchers and high-quality, industrial technology, and, in doing so, chart a new path for pharmaceutical open innovation.

Our https://opnMe.com portal is built on three initiatives:

• Molecules to order (M2O): well-characterized pre-clinical molecules delivered free-of-charge and no strings attached. Scientists who order compounds through M2O control all foreground IP rights arising from their research. As of the end of June 2022, there were 74 probe molecules available through M2O. Molecules are actively being added to the web portal at a rate of approximately one per month, with the ultimate goal of providing open access to all high-quality tool compounds.

• Molecules for collaboration (M4C): first-in-class tool compounds provided under collaboration agreements. The main aim of the M4C program is to engage outside researchers to discover new therapeutic concepts for biological targets that have no publicly reported tool compounds. Four projects initiated through M4C have returned compounds to Boehringer Ingelheim's discovery research portfolio. Although we do not reveal the compound structure or current disease areas being pursued internally, each M4C call for proposals provides researchers with both *in vitro* and *in vivo* data profiles of the compounds, thereby allowing researchers to immediately identify whether these compounds are appropriate tools for their research projects.

• opn2EXPERTS: precisely formulated questions from Boehringer Ingelheim scientists to trigger innovative science. At the time of publication of this article 20 calls have been opened to the scientific community and 457 proposals from 53 countries have been received. Despite the pandemic this program was successfully launched in 2020 and receive excellent feedbacks from the scientific community. More than 30 collaborations between academic partners or biotechnology companies and Boehringer Ingelheim have emerged from these calls.

As a member of the SGC and co-lead of the EUbOPEN project, Boehringer Ingelheim has so far contributed four co-developed – BRD9/7: BI-9564,^45^ NSD3: BI-9321,^46^ BPTF: BI-7190,^47^ SLC9A1: BI-9627^48^ – and fifteen donated chemical probes.^49,50^

To support open science projects in earlier stages and to enable anyone interested to contribute to drug design, DrugIt^51^ – a public domain drug design game – has been created in collaboration with Vanderbilt University. Finally, Boehringer Ingelheim is a full member of the CACHE initiative, which provides unbiased, high-quality experimental data on computational hit-finding predictions with the aim to define state-of-the-art *in silico* methods for drug design. Through these open science initiatives, we believe that together we can accelerate research initiatives in areas of high unmet medical need.

### Foghorn Therapeutics

Foghorn Therapeutics joined the Target 2035 initiative this year to support open science and expand the chemical probe collection with several potent small molecule inhibitors such as FHT-2344 targeting the ATPase domains of SMARCA2 and SMARCA4, also known as BRM and BRG1, respectively.^52^ BRM and BRG1 are core members of the mammalian SWI/SNF family of chromatin remodeling complexes (also known as BAF complexes), which regulate open and closed chromatin states and control transcriptional activation and repression.^53^ They are key therapeutic targets as numerous diseases have been linked to mutations or loss of BRM/BRG1 proteins, resulting in improper BAF localization and activity. The dual BRM/BRG1 probe of the ATPase domain efficiently inactivates BAF complexes, allowing for determination of relevant phenotypic outcomes in disease models which have aberrant BAF activity. These probes will not only accelerate development of potential medicines, but also enable the academic community to further understand BAF roles, activity, and important interactions.

Foghorn additionally is actively developing heterobifunctional targeted protein degraders to therapeutic targets within the BAF complex or those which regulate chromatin and transcription. The Target 2035 initiative greatly enables the targeted protein degradation field by providing qualified chemical probes which can be used as starting points for development of heterobifunctional degraders. Foghorn seeks to support these efforts by contributing, in the coming years, additional probes which can specifically be applied to targeted protein degradation.

### Janssen

There is an urgent need to bring in new validated targets across industry as we are faced with challenges like the recent pandemic or drug resistance in cancers or anti-infectives. Robust validation *via* high-quality chemical probes is an integral part of Janssen's strategy to bring new targets into the portfolio. This aligns with the Target 2035 mission to develop pharmacological tools for each protein in the proteome. Towards this mission, Janssen has donated (in the last year) six unique chemical probes,^54^ their negative control compounds and robust data packages for ROR gamma T,^55^ PDE10a, spermine oxidase, histamine receptor H4,^56^ CK1 Delta^57^ and O-GlcNAcase,^58^ and we remain committed to enable the community with high-quality pharmacological tools. Besides well-characterized probes, Janssen's JumpstARter Library is available to researchers to help “jump-start” hit finding projects, *e.g.* though our partnership with WIPO Re:Search.^59^ The library includes a diverse collection of 80 000 high-quality drug-like small molecules and compound fragments. Janssen is also planning to profile SGC's growing library of annotated chemical probes in high content imaging assays to create biosignatures for this library of molecules. Additionally, consortia such as the SGC allows for cross-industry sharing of probes to accelerate go/no go on targets that may be of interest to Janssen and not to other pharma partners.

### MSD

Pharmacological interrogation of the proteome with highly specific chemical probes is an important part of our strategy to support target validation at MSD, which is aligned with the Target 2035 initiative. In recent years we at MSD have developed three unique chemical probes and their negative controls through a collaboration with the SGC: MRK-740 targeting PRDM9,^60^ MRK-952 targeting NUDT5,^61^ and MRK-990 targeting PRMT9/5.^62^ Additionally, we have donated several potent and selective inhibitors, with their respective data packages and negative controls, from our internal collections to the community.^29,50^ MSD has also contributed spectrum-selective kinase inhibitors to aid the expansion of the PKIS sets.^63^ For the future we remain committed to promoting the use of high-quality pharmacological tools in the scientific community and will continue to enable the goals of the Target 2035 initiative through our scientific publications and disclosures of novel tool compounds with robust data packages, to support target validation.

### Merck KGaA Darmstadt

Merck KGaA Darmstadt sees a continuing need for high-quality chemical probes being made accessible to the scientific community as we are concerned about the still high number of target validation studies employing poorly characterized chemical probes. Resulting incorrect conclusions about the link between a target and a disease phenotype led to misallocation of research time and budget into drug discovery projects which likely will never provide benefit to patients. While during the recent decade the principles of high-quality chemical probes have been embraced by a part of the drug discovery community, we strongly believe that further efforts are necessary to reach deeper into the broader cell biology community.

Therefore, we are following several approaches to contribute to the Target 2035 mission. We are actively pursuing programs to identify such high-quality chemical probes together with the SGC teams in Frankfurt and Toronto. For example, we recently jointly identified MSC2711186,^64^ a SRPK1/2/3 inhibitor, which will now allow researchers to explore these understudied members of the kinome. In addition, we are encouraging our internal research teams to publish high-quality chemical probes from internal programs for those targets where we believe that target biology understanding is still limited. Towards this end we recently published MSC-4381,^65^ a highly selective inhibitor of monocarboxylate transporter 4 (MCT4) with excellent properties for cellular as well as *in vivo* studies.^66^ This probe will allow academic labs to delineate contributions of MCT4 to lactate transport for example in the context of metabolic adaptions of tumor cells. Even more recently we disclosed MSC-4106, a valuable *in vivo* usable inhibitor of the Yap/TEAD interaction.^67^ This interaction plays a crucial role in the developmental Hippo pathway which is deregulated in certain human tumors. Until now, no well characterized probe to study the role of TEAD transcription factors was available in the public domain. We believe that while some target families are already well served with high-quality chemical probes (*e.g.* certain epigenetic protein families), there are also protein families which urgently need an orchestrated effort to provide such tools. Examples include DNA/RNA helicases, metallonucleases and E3 ligases.

The Merck open innovation portal offers a series of touchpoints to the scientific community to interact with Merck and to engage in one of the many open innovation offers such as *e.g.* a training program for students from all over the world (https://www.emdgroup.com/en/research/open-innovation/innovation-cup.html), an opportunity to apply for Merck Research Grants (https://researchgrants.merckgroup.com), or an opportunity to get Merck compounds for research.

### Novo Nordisk Compound Sharing

The Novo Nordisk Compound Sharing platform^40^ was launched in 2020 to provide easy access to high-quality peptide and protein analogues many of which are not readily available to the scientific community. The intention with this open innovation initiative is to accelerate innovation. Sharing of compounds and knowledge that can lead to new findings is the key to discovering new modes of action and a better understanding of disease biology. Many academic groups, but also small biotech companies around the world have already tested novel scientific hypotheses that expands beyond the known biology of the compounds and are free to publish their data to the benefit of the entire scientific community.^68^ The list of shared compounds arises from Novo Nordisk research projects and currently comprises 20 compounds plus reference compounds including a selection of antibodies and small molecules – and the list is continuously expanded. The compounds are well-characterized and available *in vitro* and *in vivo* data as well as structural properties, handling instructions and other relevant information is readily provided on the website to ensure high-quality experiments; see an example data package for long-acting amylin analogue.^69^

Besides sharing of compounds, Novo Nordisk is also involved in other open innovation initiatives such as innovation challenges and public–private partnerships.^70^ Ultimately, open innovation will contribute to accelerating novel ideas into future breakthroughs for medical solutions.

### Pfizer

Taken alongside functional genomics, chemical probes shared with the scientific community can spare biological resources and accelerate the next steps for early drug targets. From the outset of Pfizer's participation and collaboration with SGC and EUbOPEN, the mission has been to develop high-quality tool compounds and negative controls that are free from restriction on use. We have 22 chemical probes that have so far been co-developed or donated.^71^ The initial focus of chemical probe development (*ca.* 2009) was on epigenetic targets. Of the initial epigenetic chemical probes, PFI-3 stands out as a molecule that invalidated the BRG1/BRM bromodomain within the catalytic subunit of the SWI/SNF complex as a potential drug target^72^ while the SMARCA2/4 ATPase was shown to be more fruitful.^72,73^ However, PFI-3 confirmed that a ligandable binding site in BRG1/BRM bromodomain existed which went on to catalyze further hypotheses around degradation of sub-unit components;^74^ ATPase domain inhibitors have also followed.^75,76^

More recently the gene family focus has extended into regulation of protein abundance, for example, E3 ligases and deubiquitinases. In a pilot project on E3s, Pfizer collaborated with the SGC to develop a chemical probe PFI-7 and a handle PFI-E3H1 for GID4,^77^ a subunit of the CTLH complex.^78^ The chemical and 3D structures, *in vitro* and cell-based potency, and approved vectors for synthesizing proximity pharmacology reagents were deposited in the public domain in September 2021. A recent publication disclosed another potent GID4 chemical tool which has a similar binding pose to PFI-7.^79^ Given the success with this pilot, SGC and EUbOPEN partners have set out to identify chemical matter that can extend the pool of E3s capable of inducing protein degradation through chimeric molecules, beyond the well-known cereblon and VHL state-of-the-art. Further discovered and donated probes^29,50^ from Pfizer will follow throughout the duration of EUbOPEN and beyond.

### Takeda

Takeda has been an active partner of open science pre-competitive alliances for more than a decade. The initial focus on generating meaningful chemical probes for understudied targets focused on epigenetic targets, notably protein arginine methyltransferases,^80,81^ and bromodomains.^82,83^ More recently, Takeda's efforts have focused on expanding our understanding of the “dark” kinome *via* distribution of Takeda's bespoke library of kinase inhibitors to the kinase chemogenomic set (KCGS).^63^ Takeda has provided potent tool agents to probe pharmacology of several important drug targets, with unrestricted use for academic research. Some notable ones include GPR40 antagonist, glutamate receptor NMDA type subunit 2A,^84^ O-GlcNAcase inhibitor,^85^ RIPK1 kinase inhibitor,^86^ MetAP2 inhibitor,^87^ BCL6 inhibitor,^88^ kisspeptin receptor agonist,^89^ matrix metalloproteinase 13 inhibitor,^90^ and antioxidant response element activator.^91^ The aspiration behind this initiative is to engage broader scientific community in expanding the biological & pharmacological knowledge around these protein targets and elucidate their potential implication in human disease.

Often surprising, serendipitous breakthroughs occur due to unbiased (yet non phenotypic) screening with such chemical probes if they are available. One example was recently exemplified where a pan CLK kinase chemical probe, known as T3,^92^ demonstrated robust effects on splicing regulations and efficacy in multiple oncology contexts.

While most inhibitors were developed internally as part of legacy discovery programs, Takeda has also directly participated in joint academic collaborations in open science which have led to multiple chemical probes; one notable example is the SGC3027 probe for PRMT7.^81^ Takeda remains committed to advancement of under explored areas of biology and will continue to be an active participant of EUbOPEN and similar open science pre-competitive partnerships.

### Outlook

**Fig. 2:**
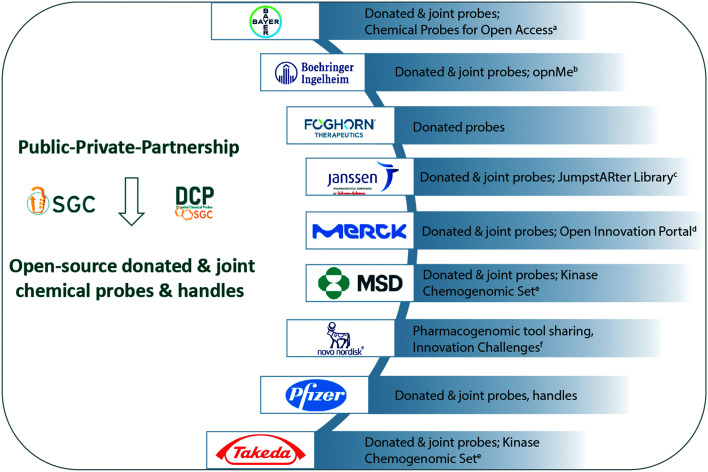
Private sector contributions to Open Science. The resources described by industry are summarized. Industry hosts a diverse set of open innovation programs, co-develop and donate pharmacogenomic tools, and often conduct off-target profiling. All chemical probes related to the SGC are listed here https://www.thesgc.org/chemical-probes, while the donated probes are listed here https://www.sgc-ffm.uni-frankfurt.de/. Between 1st May 2020 and 30th April 2025, the co-developed and donated probes and ligands will also be hosted on dedicated EUbOPEN web-pages^g,h^. Where relevant the links to commercial suppliers are listed. ^a^https://www.bayer.com/en/pharma/chemical-probes-open-access; ^b^https://opnme.com/; ^c^https://www.wipo.int/edocs/pubdocs/en/wipo_pub_research_storybook_2019.pdf; ^d^https://www.emdgroup.com/en/research/open-innovation.html; ^e^https://ximbio.com/reagent/157681/the-kinase-chemogenomic-set-kcgs; ^f^https://www.novonordisk.com/partnering-and-open-innovation/open-innovation/innovation-challenges.html; ^g^https://www.eubopen.org/chemical-probes, ^h^https://www.eubopen.org/chemical-handles.

The opinions shared by industry highlight the commitment to open science, sharing knowledge and pharmacological tools. Industry's contributions have been crucial to the success of large PPP projects such as IMI-funded ULTRA-DD (https://ultra-dd.org), ReSOLUTE (https://re-solute.eu) and EUbOPEN (https://eubopen.org). In addition, pharmaceutical companies independently host unique, open innovation programs that cater to all levels of scientists from students to entrepreneurs. During the past year, the two global initiatives CACHE^17^ and OCN^18^ would not have been launched or be sustainable without the support and insight from the pharmaceutical industry. A graphical snapshot of the open-source tools and resources described in this article are summarized (Fig. 2).

In the coming years, Target 2035 aims to continue to monitor and benchmark rapidly evolving technologies such as computer-aided drug design (CADD),^93,94^ DNA-encoded library screening followed by machine learning,^95,96^ affinity selection-mass spectrometry,^97^ automated chemical synthesis,^98^ and emerging applications of these technologies^99–103^ especially through the guidance of the private and public sectors. PPPs are a key component in developing faster, cost-effective methods to identify hits to novel targets thus accelerating the discovery of pharmacological modulators. While the private sector is predominantly focused on targets with a known disease link, government funding often supports research on a broader range of understudied proteins and protein families. For example, Genome Canada funded a two-year pilot to create drug discovery enabling reagents and know-how for the understudied WDR domain protein family. Likewise, EU funding supported the project EUbOPEN to find tool compounds for 1000 targets including targets of the ubiquitin pathway.

We hope that these accounts encourage all scientists, particularly those in the drug discovery industry, to contribute to open science projects including Target 2035.

## Abbreviations

Critical Assessment of Computational Hit-finding Experiments: CACHE
Computer-Assisted Drug Design: CADD
Enabling and unlocking Biology in the Open: EUbOPEN
Innovative Health Initiative: IHI
Innovative Medicines Initiative: IMI
Open Chemistry Networks: OCN
Public–private partnership: PPP
Structural Genomics Consortium: SGC
Unrestricted Leveraging of Targets for Research Advancement and Drug Discovery: ULTRA-DD
WD40 repeat: WDR

## Conflicts of interest

There are no conflicts to declare.

## Supplementary Material
